# Rheumatoid nodule of the thyrohyoid membrane: a case report

**DOI:** 10.1186/1752-1947-1-123

**Published:** 2007-10-31

**Authors:** Tahwinder Upile, Waseem Jerjes, Fabian Sipaul, Sandeep Singh, Colin Hopper, Anthony Wright, Ann Sandison

**Affiliations:** 1 The Ear Institute, University College London, UK; 2 Department of Head & Neck Surgery, Charing Cross Hospital, UK; 3 Department of Head & Neck Surgery, The Professorial Unit, The Royal National Throat, Nose and Ear Hospital, UK; 4 Oral & Maxillofacial Surgery/Head & Neck Unit, University College London Hospital, UK; 5 Department of Surgery, Royal Free & University College Medical School, UK; 6 Unit of Oral & Maxillofacial Surgery, Division of Maxillofacial, Diagnostic, Medical and Surgical Sciences, UCL Eastman Dental Institute, UK; 7 Department of Pathology, Charring Cross Hospital, UK

## Abstract

**Background:**

Rheumatoid nodules are common extra-articular findings occurring in 20% of rheumatoid arthritis patients. They develop most commonly subcutaneously in pressure areas (elbows and finger joints) and may occasionally affect internal organs including pleura, lungs, meninges, larynx, and in other connective tissues elsewhere in the body

**Case presentation:**

We present the case of a 62-year-old male who presented with a midline neck mass. Clinically it moved on swallowing and tongue protrusion-suggesting attachment to the thyrohyoid membrane. Ultrasound examination revealed a cystic lesion in the absence of cervical lymphadenopathy in a non-smoker. The neck was explored and histological examination of the excised lesion which was attached to the thyrohyoid membrane revealed a rheumatoid nodule.

**Conclusion:**

A rheumatoid nodule of the thyrohyoid membrane is very rare. The triple diagnostic scheme of clinical examination supplemented with ultrasound and guided fine needle aspiration for neck lumps remains valid in most cases. If excision is indicated we feel it should be performed in such a manner that the scar tract could easily be encompassed in a neck dissection excision should definitive histological examination be adverse. We suggest that when dealing with patients with established rheumatoid arthritis one should consider a rheumatoid nodule as a differential diagnosis for any swelling on the patient whether it be subcutaneous or deep.

## Introduction

Rheumatoid nodules commonly occur on the extensor surface of the forearms, in the olecranon bursa, over joints and over pressure points [1]. In almost all cases, they occur in patients with established rheumatoid arthritis and occasionally systemic lupus erythematosus.

Benign rheumatoid nodules however, can occur usually in healthy young people with no evidence of rheumatoid arthritis or systemic lupus erythematosus [2].

Cases have been reported of rheumatoid nodules discovered at unusual sites which include upper eyelid, distal region of soles, vulva and internally in the gallbladder, lung, heart valves, larynx and spine. In some patients rheumatoid nodules were first detected in these sites [3].

We report a case of rheumatoid nodule found in another unusual site namely attached to the body of the hyoid bone.

## Case presentation

A 62-year-old man with known rheumatoid arthritis on gold injection and indomethacin presented to the outpatient department with a painless swelling over left side of neck below the chin, which he noticed for the last 3 weeks. There was no history of dysphagia, hoarseness, and respiratory difficulty or weight loss. There was no other swelling in the head and neck region. The man was a non-smoker who did not take alcohol.

**Figure 1:**
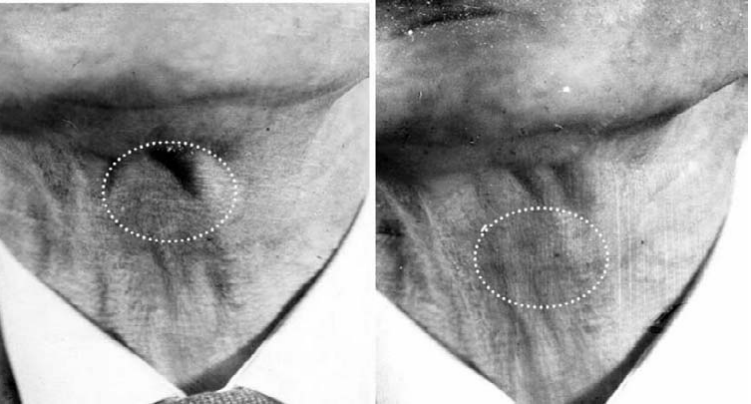
Showing movement of the lump on swallowing. Lesion outlined by interrupted line.

On physical examination, a swelling the size of a grape fruit can be seen over the superior region of the left thyroid cartilage lamina. It was non tender, felt cystic, non fluctuant, and did not transilluminate. It enlarged when he blowed up his cheeks and it moved when he swallowed and also on tongue protrusion. He did not have cervical lymphadenopathy and examination of his pharynx and indirect laryngoscopy revealed no abnormalities. Ultrasound suggested that it was fluid filled (Figure 1).

At this point, the main benign differential diagnoses included a laryngocoele, thyroglossal cyst, plunging ranulae, simple cyst or lipoma. Exploration and excision was recommended and subsequently undertaken.

**Figure 2:**
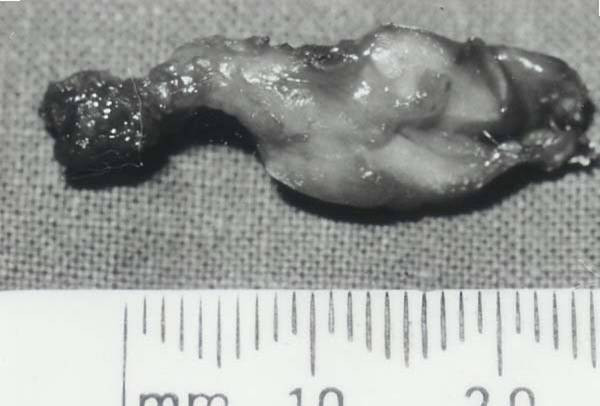
Excised operative specimen.

**Figure 3:**
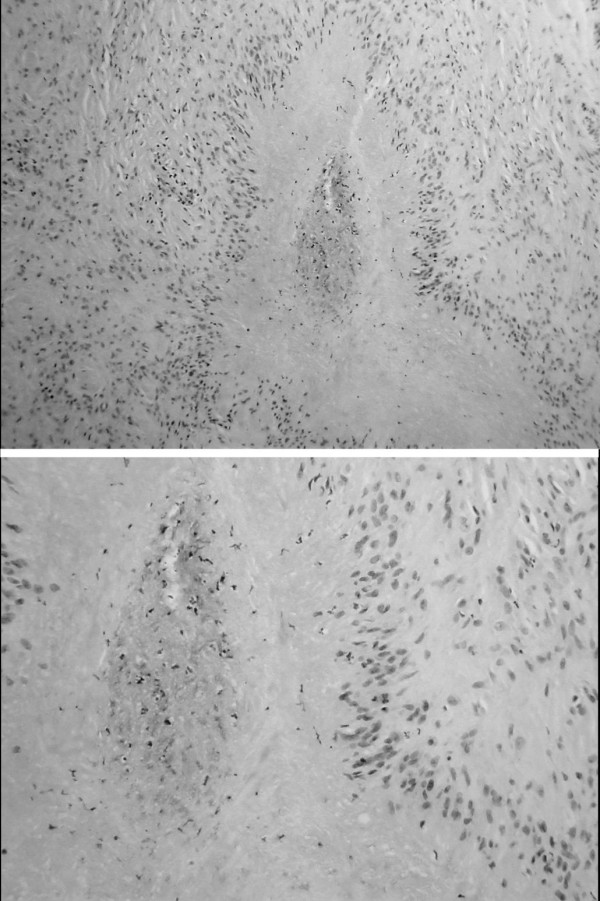
Rheumatoid nodule, ×40 magnification-top and ×100 magnification-bottom.

At surgery, a soft rounded mass the size of a grape fruit was found deep to the thyrohyoid muscle. This was dissected all round and a pedicle from the mass was traced extending to the deep surface of the body of the hyoid bone. The hyoid bone was sheared and the pedicle divided (Figure 2). The histology report stated that the sample showed dense fibrous tissue with multiple granulomata composed of pallisading epitheliod cells surrounding areas of fibrinoid necrosis. There were no giant cells and special stains for tubercle bacilli were negative. The appearance of the lesion was that of a rheumatoid nodule (Figure 3).

Patient made an uneventful recovery and follow up a year later showed no recurrence.

## Discussion

The patient was asymptomatic from the swelling but was obviously very concerned. Clinically it did not appear to be malignant. As expected, the patient's rheumatoid arthritis was active at the time and interestingly there were no obvious rheumatoid nodules found elsewhere.

A rheumatoid nodule of the thyrohyoid membrane is very rare [1]. Patients with rheumatoid arthritis may develop extra-articular subcutaneous nodules as part of the systemic disease or as initial manifestation [2]. Extra-articular manifestations are numerous, involving multiple organ systems. Rheumatoid nodules are common extra-articular findings occurring in 20% RA patients. They develop most commonly subcutaneously in pressure areas (elbows and finger joints) and may occasionally affect internal organs including pleura, lungs, meninges, larynx, and in other connective tissues elsewhere in the body [2].

In this case, the nodule was traced to the thyrohyoid membrane; however, this does not preclude microscopic attachment to one of the intrinsic joints of the larynx undergoing rheumatoid degeneration. The fibrous attachment may occur either around the cartilages of the larynx or through the anatomical points of egress of blood vessels. This is because even the small joints of the larynx may be involved by the full spectrum of pathologic changes and that both cricothyroid and cricoarytenoid joints are equally prone to the various stages of inflammation, joint destruction and ankylosis that characterize the rheumatoid arthritis elsewhere [3].

These lesions may represent significant diagnostic dilemmas in patients with clinical suspicion of malignancy. In this setting, fine-needle aspiration (FNA) of the nodules may be the simplest and most appropriate diagnostic approach [4].

The triple diagnostic scheme of clinical examination supplemented with ultrasound and guided fine needle aspiration for neck lumps remains valid in most cases. If excision is indicated we feel it should be performed in such a manner that the scar tract could easily be encompassed in a neck dissection excision should definitive histological examination be adverse.

As in this case the microscopic appearance of these 'extra-articular' rheumatoid nodules is varied [5,6]. Necrotizing granulomatous nodules are a common feature in patients with rheumatoid disease, affecting 20 per cent of seropositive patients [5].

Although there are no clinical clues that would lead to the preoperative diagnosis of rheumatoid nodule of the vocal cord and larynx, the index of suspicion should be high in patients with rheumatoid arthritis who are hoarse. However, overt joint symptoms do not appear to be a necessary concomitant of these lesions [6].

## Conclusion

We suggest that when dealing with patients with established rheumatoid arthritis or systemic lupus erythematosis, one should consider a rheumatoid nodule as a differential diagnosis for any swelling on the patient whether it be subcutaneous or deep.

## Consent

Written informed consent was obtained from the patient for publication of this case report and any accompanying images. A copy of the written consent is available for review by the Editor-in-Chief of this journal.

## Competing interests

The author(s) declare that they have no competing interests.

## Authors' contributions

**TU: **contributed to conception and design, carried out the literature research, manuscript preparation and manuscript review.

**WJ: **contributed to conception and design, carried out the literature research, manuscript preparation and manuscript review.

**FS: **contributed to conception and design, carried out the literature research, manuscript preparation and manuscript review.

**SS: **contributed to conception and design, carried out the literature research, manuscript preparation and manuscript review.

**CH: **contributed to conception and design, carried out the literature research, manuscript preparation and manuscript review.

**AW: **contributed to conception and design, carried out the literature research, manuscript preparation and manuscript review.

**AS: **contributed to conception and design, carried out the literature research, manuscript preparation and manuscript review.

All authors read and approved the final manuscript.
